# SARS-CoV-2 infection of phagocytic immune cells and COVID-19 pathology: Antibody-dependent as well as independent cell entry

**DOI:** 10.3389/fimmu.2022.1050478

**Published:** 2022-12-01

**Authors:** Olga Matveeva, Yury Nechipurenko, Denis Lagutkin, Yegor E. Yegorov, Julia Kzhyshkowska

**Affiliations:** ^1^ Sendai Viralytics LLC, Acton, MA, United States; ^2^ Engelhardt Institute of Molecular Biology, Russian Academy of Sciences, Moscow, Russia; ^3^ National Medical Research Center of Phthisiopulmonology and Infectious Diseases under the Ministry of Health of the Russian Federation, Moscow, Russia; ^4^ Institute of Transfusion Medicine and Immunology, Mannheim Institute for Innate Immunoscience (MI3), Medical Faculty Mannheim, Heidelberg University, Heidelberg, Germany; ^5^ German Red Cross Blood Service Baden-Württemberg – Hessen, Mannheim, Germany; ^6^ Laboratory of Translational Cellular and Molecular Biomedicine, Tomsk State University, Tomsk, Russia

**Keywords:** SARS-CoV-2, ADE, immune cells infection, phagocyte infection, macrophage infection, monocyte infection, pyroptosis, COVID-19 pathogenesis

## Abstract

Our review summarizes the evidence that COVID-19 can be complicated by SARS-CoV-2 infection of immune cells. This evidence is widespread and accumulating at an increasing rate. Research teams from around the world, studying primary and established cell cultures, animal models, and analyzing autopsy material from COVID-19 deceased patients, are seeing the same thing, namely that some immune cells are infected or capable of being infected with the virus. Human cells most vulnerable to infection include both professional phagocytes, such as monocytes, macrophages, and dendritic cells, as well as nonprofessional phagocytes, such as B-cells. Convincing evidence has accumulated to suggest that the virus can infect monocytes and macrophages, while data on infection of dendritic cells and B-cells are still scarce. Viral infection of immune cells can occur directly through cell receptors, but it can also be mediated or enhanced by antibodies through the Fc gamma receptors of phagocytic cells. Antibody-dependent enhancement (ADE) most likely occurs during the primary encounter with the pathogen through the first COVID-19 infection rather than during the second encounter, which is characteristic of ADE caused by other viruses. Highly fucosylated antibodies of vaccinees seems to be incapable of causing ADE, whereas afucosylated antibodies of persons with acute primary infection or convalescents are capable. SARS-CoV-2 entry into immune cells can lead to an abortive infection followed by host cell pyroptosis, and a massive inflammatory cascade. This scenario has the most experimental evidence. Other scenarios are also possible, for which the evidence base is not yet as extensive, namely productive infection of immune cells or trans-infection of other non-immune permissive cells. The chance of a latent infection cannot be ruled out either.

## Introduction

The SARS-CoV-2 virus has brought many surprises to the scientific community. One of them is the virus’ ability to infect immune cells. Numerous and rapidly growing evidence of this ability inspired us to write this literature review. Studies accumulated to date show that among immune cells SARS-CoV-2 predominantly infects professional and nonprofessional phagocytes. The observation that infecting cells are mostly phagocytes may reflect the fact that they are simply at the forefront of the body’s defense against viral infection. These cells engage various mechanisms of pathogen internalization and, in particular, phagocytosis.

Evolution has equipped some immune cells with the ability to use various variants of pathogen internalization by endocytosis, as a defense mechanism to destroy pathogens, and to utilize their peptides for antigen presentation. Phagocytosis is defined as the internalization process of particles larger than 0.5 µm in diameter (1). This process is the most ancient form of endocytosis, and the origin of proteins involved in this type of endocytic pathway can be traced back to prokaryotes (2, 3). The act of phagocytosis is a fundamental process that is playing a key role in immunity (4). Phagocytosis can be accompanied by activation of the inflammatory pathway, which contributes to the elimination of the pathogen by inhibiting its spread (5).

The cell that carries out the process of phagocytosis in an animal or human body is called a phagocyte. Phagocytic cells consist of many subsets that perform highly diverse functions to maintain our health. At the same time, these cells also play a role in pathology of infectious or endogenous nature (1, 6). Several types of immune system cells are professional phagocytes, such as neutrophils, macrophages, and dendritic cells (1, 6, 7). There are also cells that can be called nonprofessional phagocytes. For example, B-cells mainly internalize antigens through endocytosis (8), but there is some evidence that these cells have an active phagocytic capability and undergo immune activation upon phagocytosis (9, 10).

Infection of phagocytes with a virus can lead to serious malfunction of these cells, greatly undermining the protective role of the immune system. Our review focused on phagocytes, but we know that viruses capable of infecting phagocytes can be internalized by host cells not necessarily by phagocytosis. Thus, some viruses have learned to take advantage of different variants of endocytic receptor-mediated internalization pathways, including but not limited by phagocytosis, to enter and infect the target cell (11–13).

Moreover, it has already been shown that SARS-CoV-2 can transfer its replication competent RNA into the host cell cytoplasm by rapid clathrin-mediated endocytosis (14). Therefore, we consider the process of virus internalization, which leads to viral infection of phagocytes, in terms of the endocytosis and, in particular, phagocytosis mechanisms.

Some viruses can reprogram biology of phagocytic cells by infecting and using them as vehicles for the spread, and/or persistence in tissues of host organisms (15). In this review, we focus on how SARS-CoV-2 hacks into endocytic pathways to penetrate and infect an immune cell.

SARS-CoV-2 belongs to the category of enveloped viruses. These viruses enter cells *via* fusion of their envelope with cellular membranes, which might be represented by outer plasma or endosomal membrane (12, 16, 17). For coronaviruses, including SARS-CoV-2, entry into endosomes and its pH dependent release from endolysosomes are key features of the host cells entry (18).

SARS-CoV-2 cell penetration is initiated by binding of viral S glycoprotein to host cell receptors. Angiotensin-converting enzyme-2 (ACE-2) is the most studied and well characterized SARS-CoV-2 cell entry receptor (19). The interaction of the ACE-2 receptor with the viral receptor binding domain (RBD) of the S-protein causes conformational changes in both protein subunits (S1 and S2), after which the covalent bond in S2 subunit is cleaved by the host protease, leading to fusion of the viral and cell membranes, and allowing the viral genome to enter the cell cytoplasm.

To achieve the membrane fusion two sequential cleavages of the S glycoprotein are required. The first cleavage is performed by a protease (Furin) at the S1/S2 site in the host cell during S protein biosynthesis in the infected cells. The second cleavage is performed by one of two transmembrane proteases at the S2′ cleavage site when the virus reaches the next target cell. One of these proteases is the plasma membrane serine protease TMPRSS2 and the other is the endosomal cysteine protease cathepsin L (19). Cleavage of the S2′ site by TMPRSS2 occurs on the cell surface, whereas cleavage of this site by cathepsin L happens in the endosomal compartment after ACE2-mediated endocytosis. SARS-CoV-2 virus like other coronaviruses (20) can enter and leave cells through the endolysosomal system (19). SARS-CoV-2 and some related viruses can leave this system and replicate in autophagosomes in the cytosol [reviewed in (18)].

While it was discovered that there is little to no expression of ACE-2 on most human peripheral blood immune cells it was also found that ACE-2 is highly expressed on tissue macrophages (21, 22) including alveolar macrophages, and macrophages of arteries (21, 23).

Moreover, the research group of Sefik et al. revealed that ACE2 expression was higher in infected human macrophages. Blockade of ACE2 with antibodies significantly reduced the ability of the virus to infect macrophages, suggesting that the ACE2 receptor may mediate virus entry into some human pulmonary macrophages (22).

It has been shown that monocytes can also express detectable levels of ACE2 mRNA and protein (24).

However, SARS-CoV-2 transcripts were found in some migratory dendritic cells, monocyte-derived alveolar macrophages, and tissue resident macrophages, which do not express ACE2 (25). Single-cell sequence analysis of myeloid cells positive for SARS-CoV-2 RNA showed that most of them do not express known viral entry factors (26). How does the virus get into these cells? It is likely that other than ACE2 cell entry receptors are also involved. They may play an additional role in SARS-Cov-2 penetration mechanisms into different target cells, including viral entry into immune cells (27).

It has been shown that several molecules can bind SARS-CoV-2 and function as additional receptors or coreceptors, helping the virus to attach to the cell surface. Among them C-lectin proteins such as DC-SIGN, L-SIGN (28, 29), ACRG-1 (30, 31), and transmembrane proteins: KREMEN1 (30, 31), Neuropilin-1 (32), AXL receptor tyrosine kinase (33), Basigin (34, 35), and Dipeptidyl peptidase-4 (36). A list of these potential receptors, their alternative names, and information on their expression in immune cells is presented in **Table 1**.

**Table 1:** Potential receptors and co-receptors for SARS-CoV-2 entry into immune cells other than ACE-2.

Receptor	Full name	Characteristic	Evidencefor SARS-CoV-2(reference)	Immune cells expression	
SIGLEC1(CD168)	Sialic acid binding Ig like lectin 1,Sialoadhesin	I-type lectin, cell adhesion molecule	(29)	Dendritic cells, macrophages, and monocytes	(29, 37)
DC-SIGN (CD209)	Dendritic cells-specific intercellular adhesion molecule-grabbing non integri,	C-lectin, pattern- recognition receptor	(28, 29)	Dendritic cells, macrophages	(29, 38, 39)
L-SIGN (CD209L)	Liver/lymph node-specific intercellular adhesion molecule-3-grabbing integrin			Not reported	
ACRG-1	Asialoglycoprotein receptor 1, C- lectin		(30, 31)	Peripheral Blood Monocytes	(40)
KREMEN-1	Kringle containing transmembrane protein 1	Transmembrane receptor		Neutrophils and basophils	(41)
NRP1	Neuropilin-1	Membrane-bound coreceptor to a tyrosine kinase receptor	(32)	T cell subsets, T-reg. macrophages and dendritic cells	(42)
AXL	AXL receptor tyrosine kinase	Tyrosine-protein kinase receptor	(33)	Macrophages, Dendritic cells, NK-cells	(43–45)
Basigin (BSG, EMMPRINб CD147)	Extracellular matrix metalloproteinase inducer	Multifunctional transmembrane glycoprotein	(34, 35)	Leukocytes	(46)
DPP-4 (CD26)	Dipeptidyl peptidase-4	Peptidase	(36)	Widely expressed in many types of immune cells	(47)
TLR2 (CD282)	Toll-like receptor 2	Pattern-recognition receptor	(48)	Neutrophils, monocytes, macrophages, and dendritic cells	(49)

For effective viral entry into a host cell receptor expression in this cell must be complemented by the expression of proteases capable of cleaving the S-protein, as described above. Is anything known about TMPRSS2 and cathepsin-L expression in immune cells? Devaprasad et al. have shown that “a small fraction of circulating immune cells (including dendritic cells, monocytes, T cells) in the human peripheral blood mononuclear cells of COVID-19 patients express ACE2 and TMPRSS2” (50). Cathepsin-L, like other cathepsins B and H, is expressed constitutively in most immune cells (51) and therefore most likely participates in the promotion of SARS-CoV-2 entry into these cell.

The ability to infect immune cells is related to the disease pathogenesis of many enveloped viruses, such as representatives of Orthomyxoviridae family (influenza virus), Rhabdoviridae (rabies and vesicular stomatitis virus), Flaviviridae (Dengue, Hepatitis C and Zika viruses) (15, 52). The list can be expanded further with the representatives of Togaviridae (Chikungunya virus), Herpesviridae (cytomegalovirus), Paramyxoviridae (Respiratory syncytial virus), Retroviridae (HIV-1), Coronaviridae, and many others (15).

For the Coronaviridae family the phenomenon of such infection has been also documented for alpha- (53, 54) and beta coronaviruses (55–59). Some of the viruses listed use phagocytic immune cells as a repository for persistence in a form of latent infection, but some viruses employ these cells for productive infection (15).

It was hypothesized early in the pandemic that certain phagocytic cells, such as monocytes and macrophages, contribute to pathological local tissue inflammation and cytokine storm in patients with COVID-19, presumably being infected with SARS-CoV-2 (60). Subsequently, the hypothesis of a link between the development of severe immunopathology in patients with COVID-19 and infection of immune cells with the virus was reinforced. Indeed, evidence has been obtained that certain professional and nonprofessional phagocytes can be infected with SARS-CoV-2 *in vivo* and *ex vivo*, and such infection appears to be associated with severe inflammation. Our review focuses on summarizing and analyzing this evidence. We examined the possible mechanisms of infection, the consequences of this infection, and its potential role in the pathogenesis of COVID-19.

## Main

### SARS-CoV-2 infection of immune cells (lines of evidence)

There are several lines of evidence demonstrating that SARS-CoV-2 can infect and replicate in professional and nonprofessional phagocytic immune cells. The detection of viral replication and translation products in an immune cell is interpreted as the presence of an infection.

The first line comes from autopsy reports, which arrived from research teams of different countries (61–66). The second line - derives from cell culture experiments (64–71). These lines of evidence are complemented by testing of blood (66), as well as bronchoalveolar lavage fluid samples from patients with COVID-19 (25) and analysis of transgenic mice model experiments (22, 67). In a few studies the titers of the infectious SARS-CoV-2 virus produced by different types of immune cells were measured (64, 70, 72, 73). In some other studies, the infectivity of the SARS-CoV-2 pseudovirus was assessed (74, 75).

**Supplemental Table 1** summarizes the results of 19 studies in which evidence of viral infection of immune cells was obtained. The table lists the types of infected cells, as well as methods and techniques for detecting infection, and whether the virus titer was determined when there was evidence of productive infection. In addition, the table provides information on studies showing antibody-dependent enhancement of SARS-CoV-2 infection of immune cells (66, 72, 74, 75).

In summary, many researchers have concluded that immune cells are vulnerable targets for SARS-CoV-2 infection.

### Location of infected immune cells in COVID-19 patients

SARS-CoV-2 infected macrophages can be found in the lungs (25, 62, 63, 66) spleen, lymph nodes (61), as well as adipose (fat) tissues (65) of deceased patients with COVID-19. Information about adipose tissue infection is reported in the preprint, the study which has not been peer-reviewed (65). Infected cells were detected among macrophages from bronchoalveolar lavage fluid samples (25) and among blood monocytes taken from COVID-19 patients (66).

### Type of immune cells that got infected by SARS-CoV-2

Immune cells that are infected by the virus are mainly represented by professional and nonprofessional phagocytes. The first category includes monocytes, macrophages, and dendritic cells. The second category includes B-cells.

Let’s take a closer look at the representatives of cells, for which SARS-CoV-2 infection was detected. The studies described below most often point to an abortive viral infection, which, however, triggers the production of many pro-inflammatory cytokines. **Supplemental Table 1** includes a brief description of the studies we discuss in this review, listing the cells for which evidence of virus infection was obtained, as well as the methods used in the studies.

#### Monocytes

Monocytes are the largest circulating cells in the peripheral blood. They are round cells with a kidney-shaped nucleus. Monocytes can be recruited into tissues and differentiate into macrophages as well as dendritic cells, which are the front line of defense against invading pathogens. Monocytes are essential for functioning of both innate and adaptive immune systems. In addition to being precursors of other immune cells, they directly regulate immune pathways and are capable of phagocytosis. In the adult body monocytes differentiate out of precursors in bone marrow under the cascade of growth factors and stay in the circulation between 3 and 6 days patrolling the endothelial wall for the possible signal to be recruited into damaged tissue. Already in circulation, monocytes can perform their immune defense functions, and start a differentiation process (76). In blood monocytes are exposed to the number of circulating factors of endogenous and infectious origin and can be activated by these factors (77). The lifespan of circulating monocytes is rather short, and most of them, if they do not differentiate into other cells, undergo programmed cell death approximately a few days after leaving the bone marrow. After short term circulation in blood vessels, monocytes are genetically programmed to differentiate into tissue macrophages, monocyte derived dendritic cells; alternatively, they must undergo apoptosis or pyroptosis (76, 78, 79).

There is substantial evidence that human monocytes obtained from the blood of healthy donors can become infected with the virus *ex vivo* (56–58). In addition, infected monocytes have been found in individuals suffering from the disease (25, 56, 57). Thus, the research of Junqueira et al, show that about 6% of blood monocytes of patients with severe form of COVID-19 have evidence of replicating SARS-CoV-2 genomes and viral proteins translation (66). **Supplemental Table 1** lists the studies that have provided evidence of monocytes viral infection and the methods used in these studies.

#### Macrophages

Macrophages are a heterogeneous population of innate immune scavenging cells. They use both endocytosis and phagocytosis to provide protection against infection and unwanted-self components. These large phagocytes, discovered by Élie Metchnikoff, winner of the 1908 Nobel Prize, build an innate immune network in all human and animal tissues. In healthy status, macrophage dynamically controls tissue turn-over (80). When trauma occurs or pathogens invade, resident macrophages are first to provide anti-pathogen response and to active acute inflammation. At this stage, circulating monocytes are recruited to differentiate into dendritic cells and macrophages. Once the danger is eliminated, local macrophages will signal immune and somatic cells to resolve inflammation, heal the damage, and restore tissue homeostasis (6). The lifespan of macrophages varies from a few days to months or even years. Especially long-lived are resident macrophages (63, 76).

Although macrophages offer protection against infection by playing a key role in the immune defense system, they can cause serious immunopathology. Among the immunopathological conditions directly related to the improper functioning of macrophages, which turn from friends into life-threatening enemies, are sepsis (5), cancer (81), diabetes (82, 83), and heart disease (84).

Macrophage-mediated chronic inflammation is another immunopathological condition associated with malfunctioning macrophages, and it is amplified by hyperglycemia (85, 86) and dyslipidemia (87). Therefore, such inflammation is particularly harmful and can even become life-threatening for people with obesity (88), metabolic syndrome, and diabetes (89, 90). The question of how these chronic diseases with metabolic dysfunction particularly aggravate the course of COVID-19, leading to life-threatening complications, must be addressed (91). Perhaps knowledge of the pathological function of macrophages in chronic inflammation can help us find answers to this question.

*Ex vivo* polarized macrophages can efficiently engulf SARS-CoV-2. However, instead of being inactivated in these cells, the virus sometimes starts replicating and translating its proteins (67, 71).

According to the study of Lv at al. classically activated M1, but not alternatively activated M2 macrophages can be productively infected by SARS-CoV-2 and help spread the virus (67). In contrast, Boumaza et al. found that M1 and M2 macrophages were equally infected and concluded that macrophage polarization *in vitro* did not affect permissiveness to SARS-CoV-2 (69).

Yuichi Mitsui et al. found that one factor in the differentiation of macrophages into the type that is susceptible to viral infection is the lymphokine IL-10. This lymphokine is often found in elevated concentrations in the bloodstream of patients with severe COVID-19. Thus, due to this lymphokine, alveolar macrophages become M2c macrophages, which are susceptible to SARS-CoV-2 infection, and the infection leads to increased inflammation in the alveoli. The results of the study are published in a preprint (92). Thus, considering research results of (67, 71, 92) we cannot yet clearly say which type of macrophages (M1 or M2) are more susceptible to SARS-CoV-2 infection. This is clearly an important question, and further research should soon shed light on the problem and help find an answer.

How does the virus get into the macrophages it infects? One possible route of infection of residential lung macrophages is phagocytosis of epithelial cells infected with SARS-CoV-2 (73). However, FcγR-mediated phagocytosis of antibody-virus complexes instead of infected cells may also be involved in such infection (see below).

In the **Supplemental Table 1** a brief description of the studies, in which evidence of macrophage viral infection was obtained is given. This table lists subsets of macrophages that were found to be infected and includes their Cluster of Differentiation (CD) markers as well as the methods used in these studies.

Infected macrophages probably lose the ability to function properly, and their dysfunction may play an important role in the pathogenesis of COVID-19, which is described in more detail below. Because of the viral infection, macrophages infiltrating the lungs may be in a hyperactivated state. Such a state probably contributes to a positive feedback loop of release of proinflammatory cytokines by these macrophages and attraction of cytotoxic effector cells, increasing tissue damage at the site of infection (93). Some researchers have called the massive macrophage activation phenomenon during COVID-19, which leads to acute respiratory distress syndrome (ARDS), the “macrophage activation syndrome”. ARDS is the major cause of high fatality in SARS-CoV-2-associated pneumonia (94). It is highly likely that a viral infection of macrophages contributes to their reprogramming and leads to this pathological development.

#### Dendritic cells

Dendritic cells (DCs) have outer membranes that form protrusions looking like tree-like structures, which is why they got their name as dendritic. These cells are capable of amoeboid movements that allow them to travel in tissues, including tight junctions of epithelia. DCs enable bridging between innate and the adaptive arms of the immune system (95). The main function of these cells is the presentation of antigens to immunocompetent cells. The antigen presentation may or may not involve phagocytosis. DCs can digest pathogen proteins into peptides that are getting represented on the major histocompatibility complex molecules recognized by T cells (95). The life span of DCs depends upon their belonging to different subsets and is a subject of debate (96).

*Ex vivo* cell culturing demonstrated that monocyte derived dendritic cells (moDC) can be targets of SARS-CoV-1 (97, 98) and SARS-CoV-2 abortive infection (68). The observed infection of DCs induced the production of multiple proinflammatory cytokines capable of provoking interferon-mediated cell death (68).

SARS-CoV-2 transcripts were found in some migratory dendritic cells indicating likely viral infection (25). Larsen et al. observed that the virus can effectively infect and replicate in plasmacytoid dendritic cells (pDCs) causing their chronic activation (73). Experimental evidence suggests that the virus enters these cells *via* clathrin-mediated endocytosis (73). According to Larsen et al, chronic activation of pDCs, which is accompanied by their release of large amounts of IFN-I, can hyper-stimulate macrophages, causing a cytokine storm in patients with COVID-19 (73).

**Supplemental Table 1** includes a brief description of the studies in which evidence of dendritic cells viral infection was obtained, the results of these studies, as well as the methods used.

There is also a mechanism for dendritic cells to participate in the spread of the virus *via* the trans-dissemination pathway. DCs capable of transferring their cargo in a form of intact pathogens or protein complexes to other cells for antigen presentation. During this process, DCs transport intact viruses, from the sites of peripheral infection, in which the pathogen was encountered to the lymphoid tissues, where the cargo can be transferred to other antigen-presenting cells. This process is mediated by endocytic uptake of antigens or intact pathogens into late endosomal structures that are named multivesicular bodies/endosomes. These multivesicular endosomes are becoming exosomes, formed by “turned inside out” endosomes. After cargo uptake, multivesicular endosomes are directed to antigen processing through the lysosomal system or return to the cell surface, where they excrete out their cargo, and recycle surface receptors by returning them back to the plasma membrane [reviewed in (99)].

This process, created by evolution for efficient antigen presentation, has been hijacked by some viruses such as HIV and used for their dissimilation (99). The evidence is getting accumulated that coronaviruses employ this mechanism as well. DCs and many other immune cells express a C-type lectin receptor, namely DC-SIGN, which can bind the viral S-glycoprotein glycans (29). This binding possibly allows replication-competent SARS-CoV-2 to travel as a passenger with C-lectin-expressing cells, probably in multivesicular endosomes, without undergoing inactivation. Thus, DCs or other immune cells can transport the virus to the receptor-positive target cell, whereby the virus gains access to the permissive cell. In this way, DC-SIGN molecules on the surface of immune cells allow the virus not only to be taken up by the cells, but also to travel and gain access to other cell types, causing the so-called trans-infection. In this way, it has been demonstrated that C-type lectin receptors mediate SARS-CoV-2 trans-infection and can contribute to the spread of the virus (28, 29).

Interestingly, this spread infection mechanism has been shown for SARS-CoV-1. Experimental evidence has been obtained that S-glycoprotein-mediated virus entry into some host cells can be enhanced by DCs virus transmission *via* the viral coreceptor DC-SIGN (100).

#### B lymphocytes

B-lymphocytes, also called B-cells, are round-shaped cells that play a critical role in the functioning of the adaptive humoral immune system. They are responsible for the production of antigen-specific antibodies. B-cells internalize antigens through endocytosis (8), or phagocytosis (9, 10). Life span of B-cells depend on the category they belong to: there are short-lived B-cells with a lifespan of a few days and there are those that live years (101).

To date, two research teams have provided evidence that B-lymphocytes can be susceptible to SARS-CoV-2 infection *ex vivo* (64, 75). Pontelli et al. (64) demonstrated that the infection can be productive. Interestingly, a close relative of SARS-CoV-2 namely SARS-CoV-1 virus is capable of infecting human B-cells *in vitro*. Infection occurs through antibody mediated FcR-dependent infection route (102).

**Supplemental Table 1** includes a brief description of the studies in which evidence of B-cells viral infection was obtained, their results, as well as the methods used in these studies.

### Maintaining replication competence after internalization into the phagocyte

Phagocytic immune cells, including macrophages and monocytes, have an effective system for inactivating ingested pathogens. During internalization, the pathogen is captured by the cell into the endosome/phagosome, which then, together with the captured pathogen inside, undergoes several changes that include ‘maturation’ steps, with the internal pH gradually decreasing, making it more and more acidic. These steps represent a chain of fusion and fission events with endocytic organelles through a “kiss-and-run” mechanism to obtain the molecules needed for each endosome/phagosome stage. The stages of endosome maturation include the early, intermediate, and late phase (103, 104). At the end of the maturation process the endosome/phagosome with its contents fuses with the lysosome forming an endolysosome or, in particular phagolysosome, with acidic, hydrolytic contents, which can effectively kill and digest the pathogens consumed (104, 105).

Obviously, viruses that survive in immune cells after internalization use this important process of endosome maturation to their advantage. Some of them have developed escape mechanisms that allow not only to survive this endosome/phagosome transformation process, but also to start replication in the phagocytic cell.

Interestingly, it has been shown that the SARS-CoV-2 is internalized through the pH-dependent endocytic pathway (106, 107). Moreover, blocking transportation of the endosome and its acidification can inhibit SARS-CoV-2 infection (106, 107). To enter the permissive cell through endocytic pathway, the viral S-protein must undergo proteolytic cleavage by host membrane protease cathepsin L along with conformational changes. The fact that blocking acidification inhibits viral infection may indicate that the S protein cleavage by cathepsin L along with S protein conformational changes are pH dependent.

As mentioned in the introductory section of this review, enveloped viruses penetrate host cells by fusing their envelopes with cell membranes, which can be represented by endosomal membranes. Membrane fusion mechanisms can be of two types: pH-independent and pH-dependent (17). In the pH-dependent case, which we are more interested in, the interaction between the virus and the cell entry receptor leads to the obligatory endocytosis of the virus-receptor complex. Then the gradual step-by-step acidification of the endosomes, during their maturation process, promotes the viral glycoprotein cleavage by host proteases, its conformational change, followed by the fusion of the viral and endosomal membranes along with the release of the viral genetic material into the host cell (17).

It is appealing to think that a similar pH dependent mechanism is used by SARS-CoV-2 to escape the endosome/phagosome during its maturation process. Most likely, the pathogen internalization pathway with gradual stepwise acidification can provide the necessary low pH to promote proteolytic cleavage along with the conformational change in the S-protein required for the fusion of the viral and endosomal membranes followed by the entry of viral genomic RNA into the host phagocytic cell.

### Infection cycles in the immune cell, trans-infection, and the Trojan horse hypothesis

At the beginning stages of COVID-19 pandemic Matthew Park wrote a short commentary entitled “Macrophages: a Trojan horse in COVID-19?”. The commentary summarized experimental evidence by Feng et al. [published as a preprint (61)] that according to Park suggests that infected by SARS-CoV-2 macrophages could contribute to viral spread, and excessive inflammation during COVID-19 (108). It is possible that infected monocytes could play a similar role. Monocytes can become important and specialized viral reservoirs distributed throughout the body that store large amounts of viral genetic material, keeping it infective.

Indeed, according to experimental evidence [reviewed in (15)] infected monocytes can cross the blood-tissue barrier and deliver virions “as specific parcels” into the central nervous system, playing the role of the “Trojan horse”, which is a known way of dissemination for HIV, HCV, HCMV and Japanese encephalitis viruses.

However, to use the immune cell as a vehicle for spreading infection, the virus must undergo a productive infectious cycle or, in the case of the virus persistence, its infectivity in the host immune cell must be maintained. Indeed, several types of viruses can travel with immune cells, retaining viral replicative and infectious capacity until these immune cells meet permissive target cells that can be infected by these viruses (15). Therefore, immune cells may serve as a virus transport device.

Some immune cells express C lectin proteins such as SIGLEC1 (CD168), and/or DC-SIGN (CD209) on their surface (**Table 1**). These proteins have a high affinity for the outer glycoproteins of some viruses. As mentioned above, a subset of DCs belong to this kind of immune cells. They express DC-SIGN, which can bind the S-glycoprotein SARS-CoV-2.

The ability to bind and transport viruses through this and other mechanisms is an evolutionary acquisition of DCs for the antigen presentation process. However, this binding, sometimes allows the virus to hijack the adaptive evolutionary created mechanism, with the result that immune cells transport replication competent virus and promote the infection of other cell types (28, 29). Thus, lectin molecules on the surface of one cell type, such as immune cell, can be utilized by the virus to travel and access cells of another type, causing what is called trans-infection. Being involved in the process, immune cells may spread the virus but do not themselves become infected. These kinds of scenarios can lead not only to the spread of the virus to permissive cells, but also to severe functional abnormalities of immune cells.

Lv et al. observed the release of virions from infected alveoli macrophages *ex vivo*, and these virions were capable of infecting other cell types (67). In a humanized transgenic mouse model, M1 macrophages were found to promote lung infection with SARS-CoV-2. This research group concluded that SARS-CoV-2 can complete its life cycle in macrophages. This group's researchers strongly suspect that these immune cells are involved in spreading the infection within the tissue by acting as a vehicle (67). The suspicion that strengthens the Trojan horse hypothesis was further tested in a transgenic mouse model, in which alveolar macrophages were found to be able to promote SARS-CoV-2 infection of the murine lungs. Another research group showed in *ex vivo* cell culture experiments that the virus infects monocytes and macrophages without forming new infectious virions in these immune cells, but with retained infectivity for other target cells. This is how trans-infection occurs, when non-permissive immune cells associated with SARS-CoV-2 infect other permissive non-immune cells (109).

Many studies (64, 69, 71) demonstrate that immune cell virus infection proceeds abortively, without formation of infectious virions. Similar abortive infection of human immune cells, which included blood monocytes and lung macrophages was discovered by Junqueira et al. (66). The authors of the study found that up to 6% of blood monocytes in COVID-19 patients can be infected with SARS-CoV-2. However, the infectious virus was not detected in the cultures of infected monocytes, instead the signs of monocytes pyroptotic deaths were noticed that were consistent with observation of the study of Sefik et al. (22).

Abortive SARS-CoV-2 infection of human monocyte-derived immune cells such as macrophages and dendritic cells was also shown in research of Zheng et al. group (68). Therefore, spread of the virus by immune cells through productive infection in these cells seems unlikely, but transinfection of other target cells is probable.

Different research teams observe diverse scenarios under various experimental settings. Both the stages of myeloid-lineage cell differentiation, the vectors as well as states of their polarization and activation can create restrictive or permissive cellular conditions for the virus. For example, the type of infection, namely, whether the monocyte and its progeny can be infected productively or undergo programmed cell death in the form of pyroptosis, whether they will be an effective carrier of the intact virus to other target permissive cells or not, depends on the state of monocyte differentiation (15).

Monocytes, predominantly derived from the progenitors in the bone marrow, differentiate into macrophages or dendritic cells after circulating in the bloodstream for about 3-6 days. This differentiation is characterized by changes in the transcriptome, epigenome, metabolism, and secretome, which determine the key regulatory activities of macrophages and DCs in tissues (76, 78, 79).

**Figure 1:**
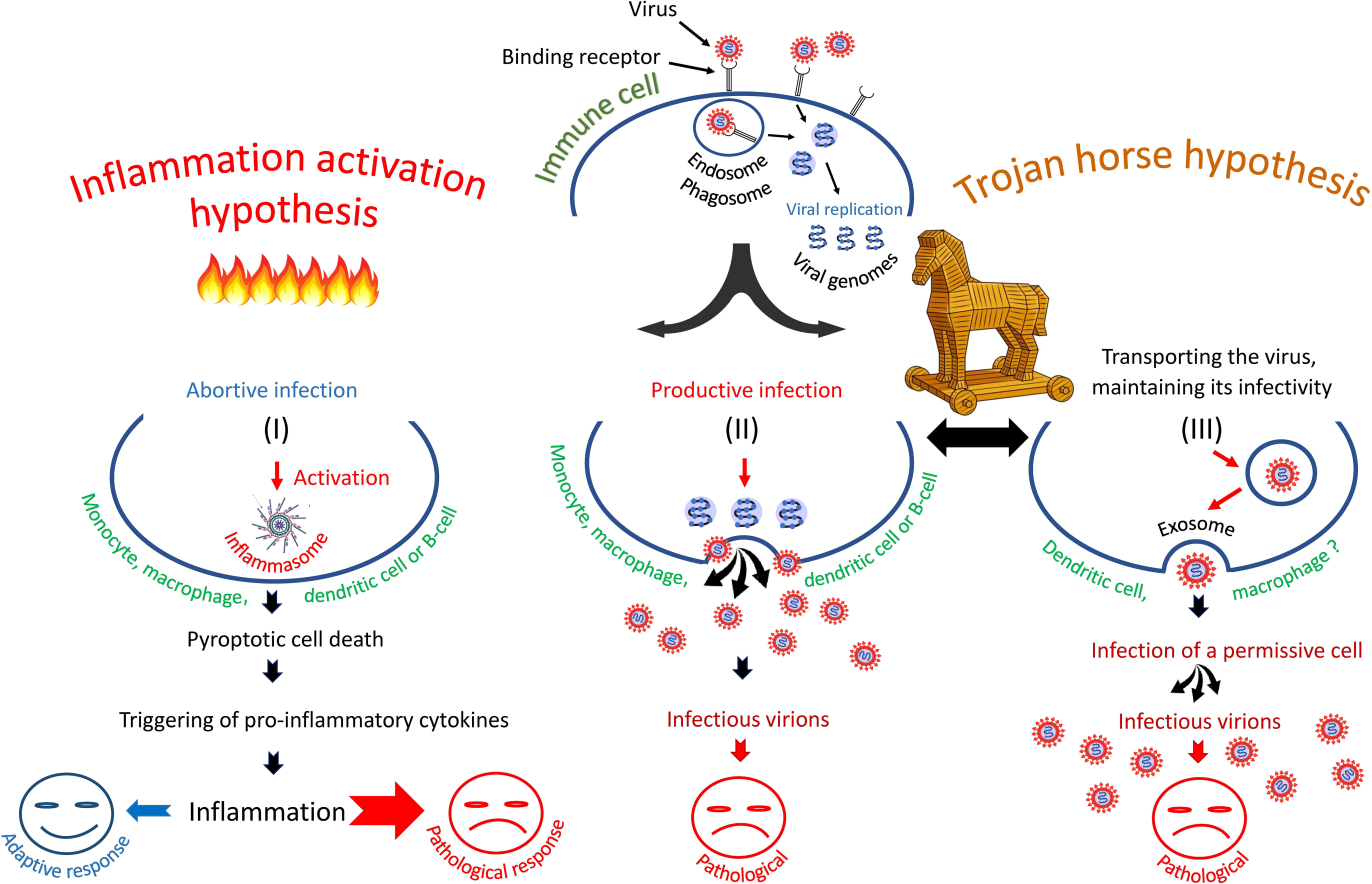
Three scenarios of SARS-CoV-2 infection of immune cells: (I) entry of SARS-CoV-2 into immune cells triggers pyroptosis and inflammatory cascade, (II) entry of virus into cells leads to productive infection or, (III) entry of virus promotes its transfer to primary permissive target cells, causing their infection. The second and third scenarios are consistent with the "Trojan horse" hypothesis.

Summarizing the experimental data one can imagine that in some immune cells the infection is abortive, in others it has a latent or productive form. There is the possibility of trans-infection of target cells by attaching the virus to the C lectins of immune cells, transporting it as a passenger on or in these cells and then infecting the target permissive cells of other type (28, 29, 109). Some possible scenarios of immune cell infection are shown in **Figure 1**.

### The role of viral infection of immune cells in the pathogenesis of disease and immune dysfunction

#### Pyroptosis as a trigger of inflammation cascade and COVID-19 pathology factor

Studies performed on transgenic humanized mice and in cell culture showed that SARS-CoV-2 replication in macrophages triggers inflammatory death (pyroptosis) of these cells, which likely stimulates the hyperinflammatory cascade associated with COVID-19 (22). Pyroptosis is a form of lytic programmed cell death that happens most frequently during infection with pathogens having the capability to enter and survive in the cells of the host organism (110, 111). This type of suicide cell death occurs mainly in immune cells such as macrophages that are responsible for the inflammatory process, however other cell types such as epithelial cells can also undergo pyroptosis (110, 112). Thus, infection-induced pyroptosis probably represents an important line of defense, carried out not only by immune cells, but also by epithelial barrier tissues.

The pyroptosis promotes the release of immunogenic cellular content, including damage-associated molecular patterns, and inflammatory cytokines such as interleukin-1β (IL-1β) (110). The cytokine release provokes inflammation, which, being part of the body’s normal immune defense mechanism against infection, can be harmful if it is dysregulated. For example, increased uncontrolled inflammation can lead to sepsis (110).

The physiological function of inflammation and pyroptosis is to trigger the expression of immune genes and attract lymphocytes to the site of infection, thereby controlling pathogen invasion (113). In addition, pyroptosis of infected cells prevents the virus from completing its replication cycle, therefore it is an important part of the antiviral immune defense mechanism (113, 114).

Not surprisingly, because of these properties of pyroptosis, the release of infectious virions by the infected macrophage is largely prohibited and was observed only after treatment of the cell with inhibitors of the inflammasome pathway, which prevent pyroptosis (66). Thus, there is no evidence of a complete viral cycle naturally occurring in macrophages in the study by Junqueira et al. (66). However, despite the lack of detection of the productive cycle of the virus, the study showed that signs of pyroptosis in the blood plasma of COVID-19 patients correlate with the development of severe disease. Therefore, Junqueira et al. concluded that abortive viral infection of macrophages, due to pyroptosis, triggers the excessive production of inflammatory cytokines, which enhances the hyperinflammatory response to SARS-CoV-2.

These findings were further supported by experimental data that the virus induces inflammasome formation and activation, which leads to pyroptosis in experimentally infected primary monocytes from healthy donors and COVID-19 patients (70).

Inflammasomes are cytosolic multiprotein complexes that are a functional part of the mechanisms of the innate immune system (113, 115). Activation of the inflammasome stimulates secretion of pro-inflammatory cytokines such as IL-1β and interleukin 18 (113, 115). Therefore, it is not surprising that SARS-CoV-2-infected blood monocytes and lung macrophages had activated inflammasomes. It has been demonstrated that these activated inflammasomes and programmed cell death *via* pyroptosis increases the secretion of proinflammatory cytokines, including IL-1ß by SARS-CoV-2-infected monocytes (70). Super elevated cytokine secretion promotes tissue immune cell infiltration, which through over secretion of proteases and reactive oxygen species can cause damage to the lung and other human organs.

To what extent does a physiological immune defense mechanism, such as pyroptosis, whose purpose, among other things, is to prevent the spread of infection, become dysfunctional because of infection with SARS-CoV-2 macrophages, monocytes, or other immune cells? To what extent does this infection contribute to the aberrant inflammatory feedback loop? These questions need answers.

The authors of the study Junqueira et al. examined whether the macrophages in the lung autopsies of diseased COVID-19 patients were infected with SARS-CoV-2 and whether they had activated inflammasomes. They found that a smaller proportion of macrophages were infected, but a larger proportion had activated inflammasomes. This ratio indicates that pyroptotic death of infected cells leads to the release of potent inflammatory mediators, causing activation of inflammasomes in the larger macrophage population (66).

The observation of this phenomenon and other evidence led to the development of the idea that viral infection of immune cells, triggering death of these cells through pyroptosis, is the massive driving force behind the development of particularly severe forms of the disease and fatal COVID-19 cases. The idea is becoming more and more popular in the research community (22, 66, 70).

#### Productive virus infection in immune cells

Lv et al. observed the release of infectious virions from infected macrophages *ex vivo* (67). Interestingly, it has been shown that the N-protein SARS-CoV-2 can antagonize cellular inflammatory responses by inhibiting pyroptosis in human monocytes (116). It remains to be shown if this inhibition can lead to a productive viral infection of immune cells.

##### Inhibition of IFN-β production may contribute to COVID-19-associated pathology

It has been demonstrated earlier that SARS-CoV-1 infected macrophages induce little or no IFN-β, suggesting that suppression of the immune response leads to uncontrolled viral replication in the respiratory epithelium cells (117). Perhaps a similar scenario is realized with SARS-CoV-2.

### Mechanism and cell entry receptors

#### Direct infection through cell entry receptors (not antibody mediated)

Infection of immune cells can occur in two ways, which are probably synergistic. That is, a cell can become infected with a virus by viral interaction with a cell receptor without assistance of antibodies or with such assistance. In the case of the latter, antibodies help to infect the immune cell. Below we will discuss these two pathways, but it should be remembered that they are not mutually exclusive and, most likely, each of these pathways of virus penetration into the cell increases the probability of infection. In the “Introduction” section, we gave a list of receptors and co-receptors (**Table 1**) that, in addition to the ACE 2 receptor, can be used by SARS-CoV-2 to penetrate immune cells.

Direct infection of primary human SARS-CoV-2 monocytes using cell entry receptors and without any antibody assistance was demonstrated by Rodrigues et al. (71). This group, like Sefik et al. (22), found that the virus can replicate in healthy donor monocytes cultured *ex vivo*, and this process causes NLRP3 inflammasome activation as well as cell death. NLRP3 is a protein mainly expressed in macrophages and is encoded by the NLRP3 gene located on human chromosome 1. The protein acts as a sensory molecule and, together with other proteins, forms the NLRP3 inflammasome, a protein complex essential to the innate immune system functioning (118).

The process of monocyte infection detected by Sefik et al. (22) and some other research teams (68) did not require antibodies for the virus cell entry. Similar finding was done by Pontelli, et al. This research team revealed that monocytes, as well as both B and T lymphocytes, were susceptible to direct SARS-CoV-2 infection *in vitro* that didn’t require antibodies. The discovery of the phenomenon raises the question related to the identification of receptors that can be used by the virus to enter the cell.

#### Antibody mediated infection route *via* Fcγ receptors

Antibody-dependent enhancement (ADE) of infection is a phenomenon in which virus-antibody complexes interact with immune cells carrying complement or Fc-receptors and promote internalization of the virus, maintaining infectivity and virus potential for latent, abortive, or productive infection (119–122). In other words, antiviral antibodies facilitate the entry of the virus into immune target cells by hijacking the phagocytic FcγR or complement pathways, which naturally evolved to fight infection. Although antibodies are usually protective and beneficial to organisms, surprisingly, they can promote a viral infection instead of stopping it. It has been shown that virion binding to non-neutralizing or sub-neutralizing antibodies can lead to more efficient FcγR mediated viral uptake by the target cell causing ADE (121).

The ADE phenomenon has been described for many viruses including representative of families *Flaviviridae (Dengue virus), Retroviridae (HIV-1), Orthomyxoviridae* (Influenza virus), *Paramyxoviridae* (Respiratory syncytial virus), *Filoviridae* (Ebola virus), and *Togaviridae* (Chikungunya virus) (122–124).

The ADE has been also documented for *Coronaviridae* including alpha- (53, 54) and beta coronaviruses (55–59). ADE could increase the severity of several viral infections (75) although no definitive role for ADE in human coronavirus infections has been demonstrated so far. However, it is well established that feline infectious peritonitis virus can replicate in monocytes and macrophages, entering these cells *via* antibody dependent mechanism. This replication can trigger fatal peritonitis in cats (53, 54). Therefore, some researchers hypothesize that the pathogenesis of SARS (56–59) and COVID-19 (55, 125–127) diseases may be associated with ADE. Its role in establishing potential latent virus infection is also discussed (55).

Interestingly, the research group of Junqueira et al. discovered that monocyte SARS-CoV-2 infection can be dependent on antiviral antibodies. It is worth noting that only antibodies from COVID-19 infected patients, and not from the plasma of vaccinated individuals, were able to contribute to the type of cellular infection observed by this group (66).

It is well established that in many viral diseases, prior sensitization of the humoral immune response, in the form of prior infection or vaccination, is a prerequisite for ADE upon subsequent antigen exposure (121). However, according to Junqueira et al. (66), for SARS-CoV-2 the scenario is different; antibody mediated virus entry can occur during primary viral infection.

Sanchez-Zuno et al. (128) suggested that currently there is no sufficient evidence that ADE promotes the spread of SARS-CoV-2 in infected host organisms. Therefore, ADE in COVID-19 is best described as “virus antibody dependent entry into the cell”, or “antibody-dependent inflammation”, which does not necessarily lead to productive viral infection, meaning that ADE can lead to abortive or latent SARS-CoV-2 types of infection instead. Other research team suggested the name Fc-mediated viral entry (FVE) (129).

However, as mentioned above, even abortive infection of immune cells can lead to powerful amplification of inflammatory cascades with aberrant feedback loops, which can promote severe form of COVID-19. A similar scenario occurs with other viruses, particularly the Dengue virus, for which it has been noted that ADE has been associated with the development of a “cytokine storm”, which involves the massive release of inflammatory cytokines and other mediators (130).

How does a virus use antibodies to enter an immune cell? The process resembles the piggybacking approach, in which “piggyback” is an IgG antibody and the entry gates to a pig barn are Fcγ receptors (FcγRs) of immune phagocytic cells (131, 132). It is thought that myeloid cells expressing FcγRs, such as monocytes and macrophages, dendritic cells, and some granulocytes, can become ADE targets through phagocytic uptake of immune complexes (128).

**Figure 2:**
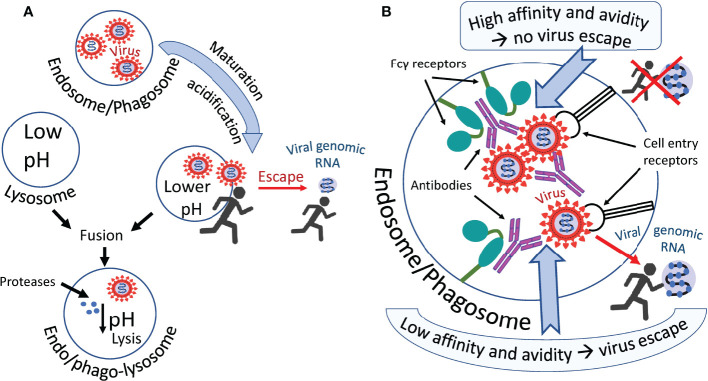
Hypothetical ways in which the virus escapes phagosomes. **(A)** The virus leaves the endosome/phagosome during its maturation and acidification. **(B)** Antibodies that firmly retain the virus in the immune complex most likely prevent it from leaving the organelle as shown in the upper part of the figure. Antibodies not firmly holding the virus allow it to escape from the organelle into the cytoplasm, as shown in the lower part of the figure. Perhaps acidification of the endosome/phagosome during its maturation reduces the affinity of some antibodies to the virus, promoting the exit of the virus from the immune complex and facilitating its exit from this organelle.

Most likely cooperation between conventional cell entry receptors such as ACE2 and Fc receptors occur and both types of receptors are working synergistically. For example, Wang et al. found that ACE2 can function as an additional receptor in the FcγR-dependent ADE of SARS-CoV-2 infection (74). A possible mechanism of antibody-mediated infection *via* Fcγ receptors is shown in **Figure 2**. The virus probably escapes the endosome/phagosome during the maturation of this organelle, which is accompanied by its acidification, as shown in **Figure 2A**. Two scenarios can be imagined of this escape (**Figure 2B**). They differ in the affinity of the antibody and the resistance of this affinity to a pH decrease in an endosome/phagosome. The first scenario: in the case of high antibody affinity, which is also unaffected by a pH decrease, the virus is reliably retained by the antibodies within the immune complex in the endosome/phagosome. It cannot escape. However, second scenario is possible: in the case of low antibody affinity, which can be further decreased with the acidification of the endosome/phagosome during its maturation, the antibodies are losing their “grasp” and the virus acquires the ability to escape from the immune complex and consequently, also the endosome/phagosome.

FcγRs belong to the family of immunoglobulin proteins. They are located on the surface of immune cells and function as molecules that bind Fc-chains of the IgG molecule (133). This binding might trigger phagocytosis of the virus-antibody complex (131). One of the natural functions of FcγRs is to help phagocytic immune cells to capture, and “ingest” the pathogen. Internalization of the pathogen should lead to its inactivation and presentation of the pathogen derived antigens by the phagocytic cell. However, some viruses have developed the ability to avoid inactivation in the immune cell.

Three FcγR groups have been described for human cell types: FcγRI (CD 64), FcγRIIA/B (CD32), and FcγRIIIA/B (CD16). All receptors are expressed in various combinations on the surface of different immune cells (133). FcγRs vary in their affinity for IgG, such as FcγRI is a high-affinity activating receptor that binds to monomeric IgG molecules, FcγRII and FcγRIII are low-affinity receptors that require high avidity binding by IgG-pathogen immune complexes. Among FcγRII receptors FcγRIIA and FcγRIIC are activating receptors and FcγRIIB is an inhibitory receptor (122, 133).

Experimental evidence suggests that SARS-CoV-1 can start replication in immune cells after entering *via* FcγRII (CD32) (56, 102, 134). It was specifically shown that the expression of two types of receptors by immune cells: FcγRIIa and FcγRIIb induces ADE by SARS-CoV-1 (56). The author of another study, while observing SARS patients, found that the severity of the disease correlates with the FcγRIIa allelic polymorphism. In patients with FcγRIIa allelic isoform that can interact with both IgG1 and IgG2, the disease was more severe compared to patients with the FcγRIIa isoform capable of binding only IgG2 (134). Thus, it appears that obtained evidence points to CD32 as the major receptor for SARS-CoV-1 entry.

It has been discovered that FcγRIIB is involved in infection of B-cells by SARS-CoV-2 (75). However, it is likely that additional receptors function in antibody-mediated immune cell entry of SARS-CoV-2 compared to SARS-CoV-1. Thus, team of Junqueira et al. found that blocking CD16 or CD64 strongly inhibited infection by SARS-CoV-2 of monocytes, whereas blocking the other receptors had no major effect (66). This experimental evidence points to CD16 or CD64 as major virus entry receptors for SARS-CoV-2.

The Fcγ receptors used by MERS-CoV, SARS-CoV-1, and SARS-C0V-2 for immune cell infection are listed in **Table 2**.

**Table 2:** FcγRs receptors involved in the antibody-dependent infection of immune cells by beta-coronaviruses.

Receptor group	Affinity to Fc region of IgG(reference)	Subgroup	Cells, expressing the receptor, among those that can be infected by a coronavirus(reference)	Virus	Cells that are targets for the virus infection *via* Fc receptor(reference)
FcγRI (CD64)	High (135)	Un specified	Macrophages, DCs (133, 135, 136), Monocytes (72)	SARS-CoV-2	Monocytes, macrophages (66)
FcγRII (CD32)	Low (135, 137)	FcγRIIa	Monocytes, Macrophages, DCs (138) (133, 135),, B-cells (72)	MERS-CoV	Model nonimmune cells, model cells transfected with FcγRIIa encoding gene and macrophages (139)
				SARS-CoV-1	B-cells-Raji (Burkitt’s lymphoma B lymphoblast), Daudi (Burkitt’s lymphoma, B lymphoblasts), Monocytes (56, 102)
		FcγRIIa	Monocytes, Macrophages, DCs (133, 135, 138)		Cells were not tested but a significant association was found between the Fcgamma RIIA-R/R131 genotype and a severe course of SARS (134)
FcγRII (CD32)	Low (135)	FcγRIIa	Monocytes,Macrophages, conventional DC (133, 135, 138)	SARS-CoV-2	Model cells transfected with FcγRIIa encoding gene (129)
				SARS-CoV-2	Model cells transfected with FcγRIIa encoding gene, established cell culture of B-cells (Raji cells) (74)
	Low (135)	FcγRIIb	B-cells, Monocytes, Macrophages, DCs (133, 135)	SARS-CoV-2	Model cells, B-lymphocytes (Raji cells) and lymphoblasts (Daudi cells) (75)
FcγRIII (CD16)	Low for fucosylated Fc fragments and high for afucusylated fragments towards CD16a (135, 140)	FcγRIIIa	Small minority of blood monocytes (10%) and resident macrophages in some tissues (66, 135, 138), B-cells (72)	SARS-CoV-2	Monocytes CD16+, CD 64+, lung macrophages (22, 66)

##### IgG afucosylation contributes to Fc-mediated virus phagocytic uptake, infection, and disease severity

Immune responses in severe COVID-19 patients stand out with a predominance of low fucosylated IgG antibodies (141). Although Fc afucosylation is supposed to be a regulatory layer of adaptive immunity (142), many studies reported its role in COVID-associated pathology (141, 143–145).

**Figure 3:**
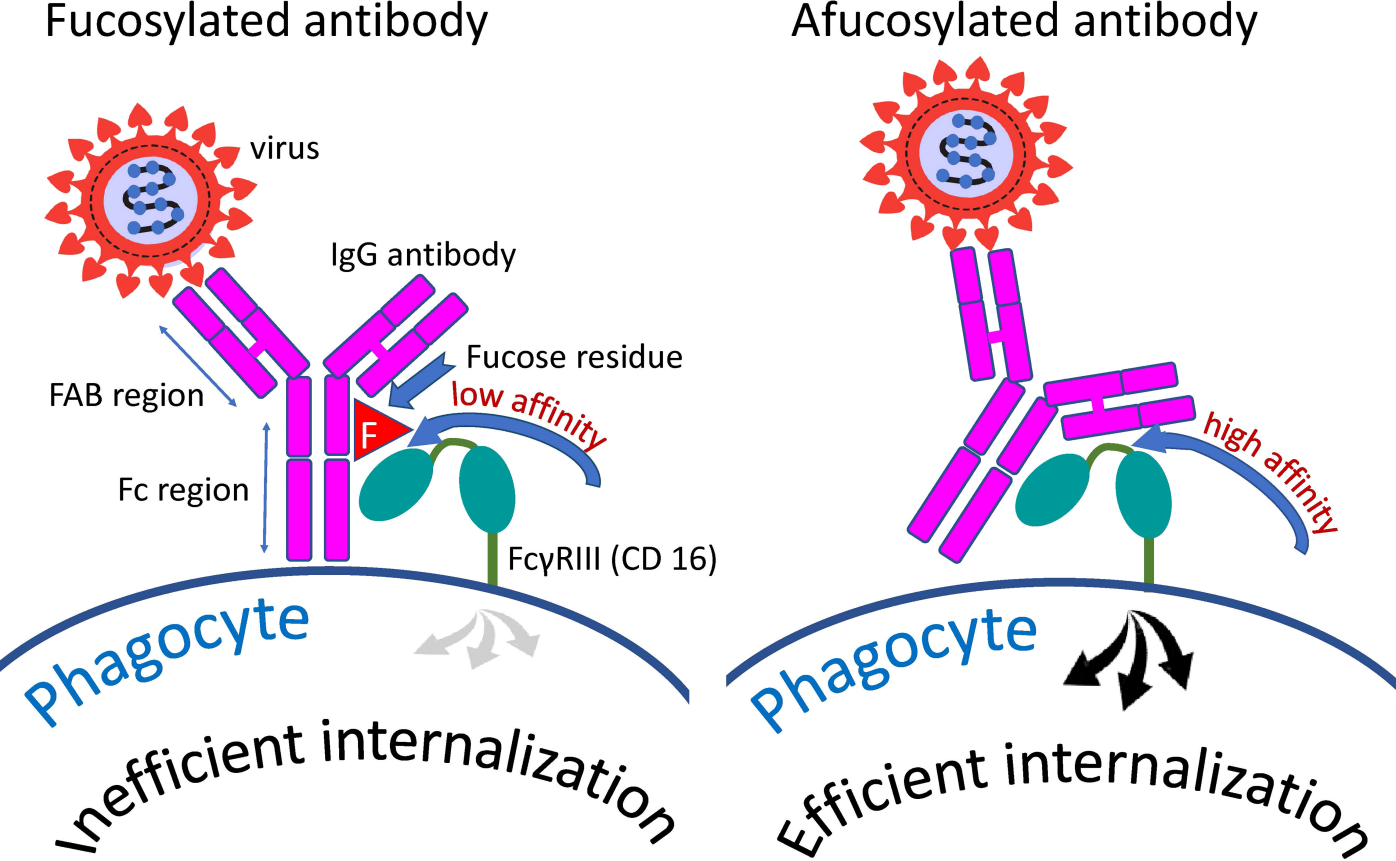
Distinction between fucosylated and afucosylated antibodies. Fucosylated antibodies have a lower affinity for the cellular receptor FcγRIIIa (CD16a) compared to afucosylated antibodies. Consequently, these antibodies contribute less to the internalization of the pathogen by cells possessing this receptor than do afucosylated antibodies.

Core fucose is a component of N-linked glycans within the Fc fragment of the antibody. Addition of fucose does not cause structural changes (146) but strongly decreases the affinity of receptor binding (147) and the efficiency of antibody-dependent reactions (148). A schematic representation of the interaction of an antibody with the phagocytic Fc gamma RIII receptor and the effect of a fucose residue on this interaction is shown in **Figure 3**. Afucosylation is not the stripping of existing fucose, but simply fucosylation that has not happened. Core afucosylation seems to be an adaptive mechanism for the response to membrane-bound antigens (142). Since afucosylated IgG are shown to have a higher receptor-binding affinity and thus more pronounced effector functions, Fc afucosylation might be a regulatory feature to adjust adaptive humoral immunity to fight enveloped pathogens. However, it can also promote autoimmune pathology (149–151) and aggravate infectious diseases (152–154).

Afucosylated IgG antibodies have been shown to contribute to the pathogenesis of COVID-19 through their proinflammatory action (141). In particular, Chakraborty et al. showed the link between low fucosylation of viral S protein-specific antibodies and the release of proinflammatory cytokines, such as IL-6 and TNFα, during SARS-CoV-2 infection (143). Another study confirmed these results and revealed that high titers of afucosylated IgG antibodies targeting S protein induce inflammation promoted by alveolar macrophages and activate platelets as well as endothelial cells (144). Authors observed the normalization of antibody fucosylation in several weeks after infection.

In their next study, Chakraborty et al. found out that afucosylated IgG antibodies were non-neutralizing and could be detected in severe COVID-19 patients but not in those with mild disease or vaccinated (145). Noteworthy, van Coillie et al. reported in their preprint that the first dose of mRNA vaccine induced transient formation of low fucosylated IgG1 in people naive to SARS-CoV-2, but not in people who encountered this antigen, although the extent of this formation was lower than in those with severe COVID-19 (155).

Immune complexes with low fucosylation from patients with severe COVID-19 induced immune cell infiltration of lung tissue in model animals, whereas highly sialylated and fucosylated mRNA vaccine-elicited IgG did not cause inflammation in lungs or proinflammatory cytokine release (145).

Junqueira et al. investigated how SARS-CoV-2 infects monocytes and observed that highly afucosylated antibodies from COVID-19 patients but not from vaccinees significantly increased virus uptake through FcγRIII (66).

Thus, there is growing evidence that IgG antibodies with afucosylated Fc fragments play an important role in the pathogenesis of viral infections and particularly in COVID-19.

##### SARS-CoV-2 entry into a cell *via* complement receptors. Complement-and antibody mediated ADE

Preliminary data indicate that a pathway other than FcγR-mediated cell entry may be involved in SARS-CoV-2 ADE. Okuya et al. obtained evidence that an antibody-dependent pathway of virus entry mediated by the complement component 1q plays a role in cell infection with this virus. However, immune cells were not investigated as target cells in this study (156).

**Figure 4:**
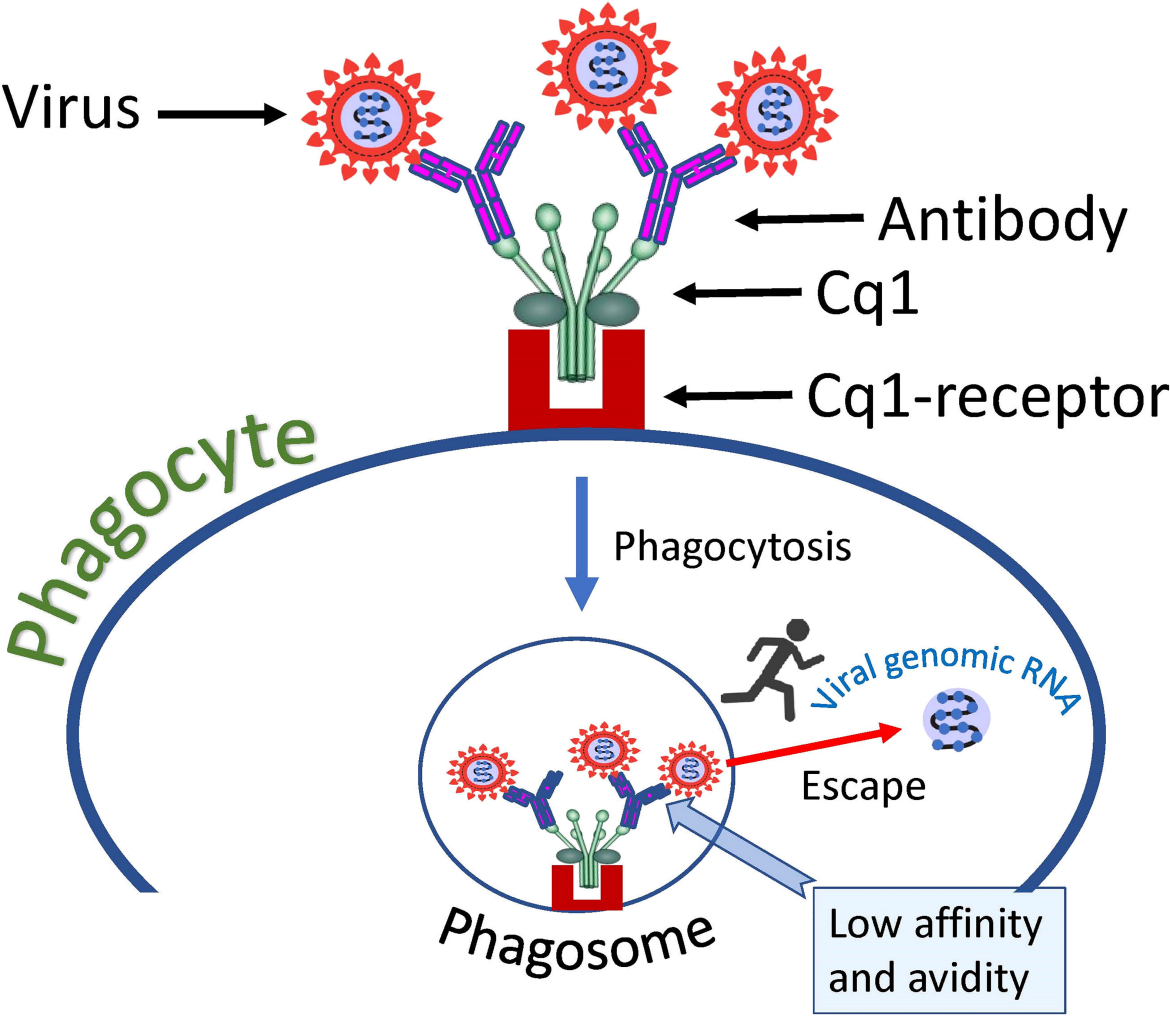
Hypothetical mechanism of SARS-CoV-2 entry into a cell *via* complement receptors. The low affinity of the antibody, which causes a loose connection to the virus, allows the virus to escape the complex and fuse its envelope to the phagosome membrane, thus introducing its genome into the cell cytoplasm.

Complement component 1q (C1q) is a protein complex involved in the bridging of innate and adaptive immune systems. Okuya et al. provide a review of the literature showing that C1q-mediated ADE has been proposed for several viruses, including HIV, EBOV, Marburg virus, and human parvoviruses (156). This ADE mechanism is based on the binding of C1q receptors on the cell surface with virus-antibody C1q complexes, which results in enhanced attachment of the virus to target cells. The mechanism is like FcγR mediated virus cell entry, but instead of FcγR another cellular receptor is used namely - C1q receptor. Therefore, C1q serves as an additional bridge connecting virus-antibody complexes to the cell plasma membrane. These complexes are then taken up by the phagocytic cell and end up in the endosome/phagosome (**Figure 4**).

Junqueira et al. observed that patient plasma promoted viral infection of healthy donor monocytes *in vitro* much more than purified IgG antibodies against S-protein (66). The authors of the study concluded that an antibody-independent plasma component may contribute to the infection (66). It is possible that C1q is the plasma component responsible for the effect observed by the study authors.

##### ADE associated antigenic determinants and antibodies: a comparison of SARS-CoV-1 and SARS-CoV-2

ADE of coronaviruses can be promoted by antibodies to the spike (S) glycoprotein. This observation was done for SARS-CoV-1 (59, 102, 157) and SARS-CoV-2 (127, 158).

A high neutralization titer of antibodies to the SARS-CoV-2 spike-protein in the plasma of patients with COVID-19 has been shown to predict survival (159). However, different antibodies have various potential to neutralize the virus and cause ADE; a neutralization potential does not exclude a potential for ADE. Anti S-protein immune serum, while inhibiting receptor mediated viral entry into a permissive host cell, may increase infection of human monocyte-derived macrophages (59) and cultured B-cells (102, 157) by SARS-CoV-1 *via* Fc-receptor binding.

Some antibodies can have both neutralizing and ADE effects. Antibodies targeting S-protein that neutralized most variants of SARS-CoV-1 viruses enhanced immune cell entry of the mutant virus. The mechanism of enhancement might involve the interaction of antibodies with conformational epitopes in the viral ACE-2-binding domain (160). Antibodies targeting different S-protein variants of the SARS-CoV-1 virus can neutralize the virus or facilitate its entry into the cell. If anti-S antibodies target a human-derived virus protein, they neutralize the virus. The same antibodies enhance virus entry into the cell if the virus has been adapted for growth in palm civet and acquired a mutated gene encoding the S-protein in the process of adaptation.

The ACE-2 receptor-binding domain of S-protein can mediate antibody-dependent virus entry. Five amino acid substitutions in the S-protein region from positions 248 to 501 in adapted human and civet viruses are probably responsible for this effect (60). Interesting, human immunodominant epitopes of the SARS-CoV-1 have been shown to cause both enhancing and neutralizing effects in non-human primates. In rhesus macaques, the S-protein peptides S471–503, S604–625, and S1164–1191 triggered antibodies that efficiently prevented infection. In contrast, peptide S597–603 elicited antibodies that enhanced infection both *in vitro* and *in vivo* (57).

Wang et al. noticed that two neutralizing monoclonal antibodies enhanced the ability of the SARS-CoV-2 pseudovirus to infect B-lymphocytes, but another neutralizing monoclonal antibody was not able to help the virus infect these cells (75). Interestingly, the antibody that was unable to cause ADE was capable to bind only “up” position of RBD of S-protein, while antibodies associated with ADE could bind to RBDs in S-trimer with both “up” and “down” states (75).

Certain epitopes of the S-protein are particularly prone to be targeted by antibodies that promote ADE. These observations were done by Zhou at al. using convalescent plasma from donors. The group revealed that enhancement versus neutralization by SARS-CoV-2 antibodies associates with distinct epitopes on the RBD of S-protein (127).

##### Effect of antibody concentration and epitope-antibody binding constant on the ADE effect

**Figure 5:**
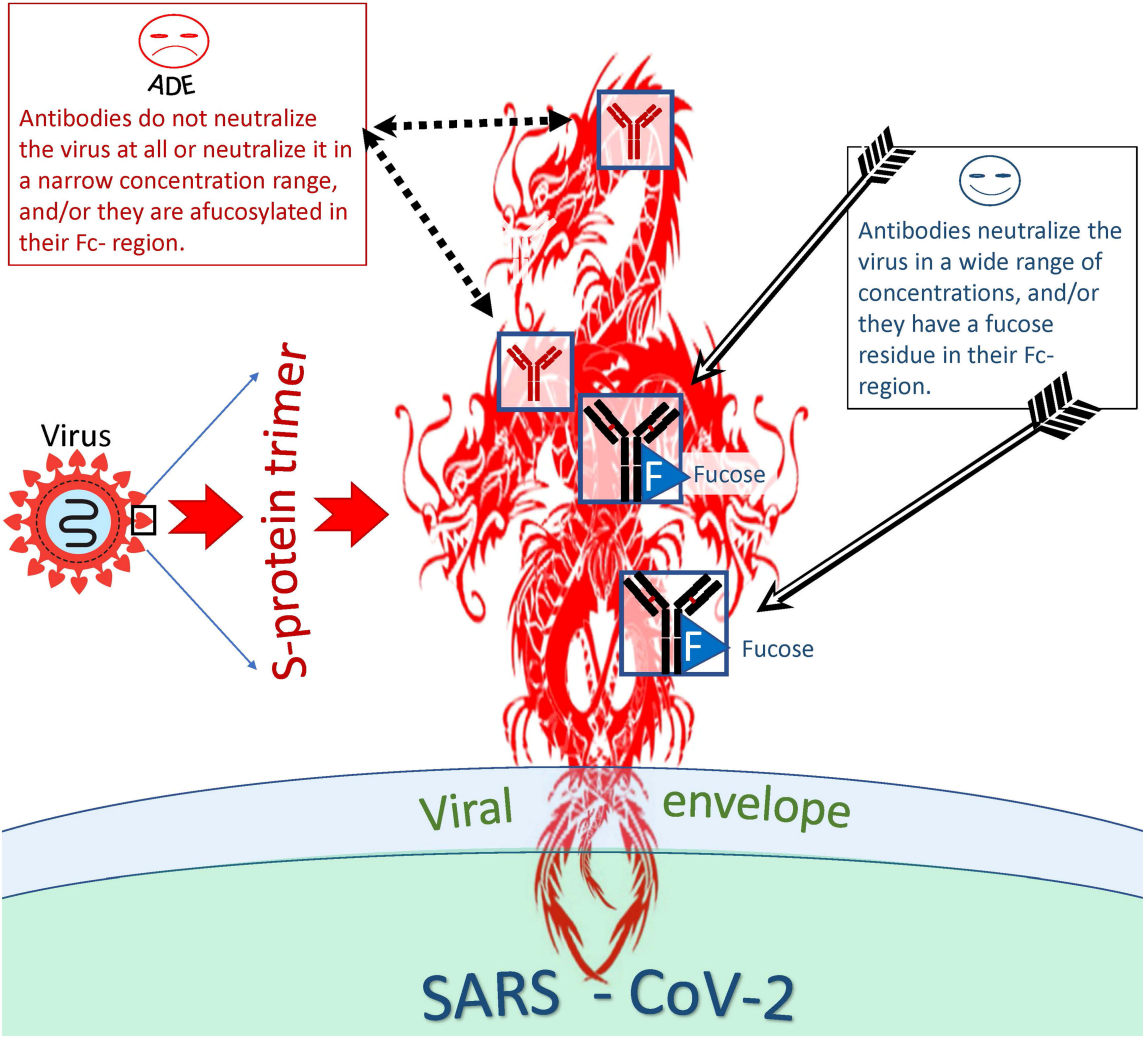
Distinction between antibodies that are more or less likely to cause ADE. Antibodies capable and incapable of causing ADEs differ both at the level of epitope recognition and by the type of antibody itself. Thus, antibodies to certain antigenic epitopes are more prone to cause ADEs than antibodies directed at other viral epitopes. In addition, afucosylated antibodies are more likely to cause ADEs compared to fucosylated antibodies. However, even antibodies capable of inducing ADE at one concentration can neutralize the virus at another and be protective against the virus.

When studying the ADE phenomenon *in vitro*, researchers noticed the so-called antibody concentration effect, which perhaps is related to the fate of the virus-antibody complex inside the phagocytic cell (157) (**Figure 5**). This effect is that some antibodies can neutralize the virus in a wide range of concentrations and others in a narrow range; they can neutralize the virus at a high concentration but help the virus to infect cells at a lower concentration. Fc-mediated SARS-CoV-2 uptake by cell (causing ADE) might peak as neutralization potency of the relevant antibody decreases (129). In other words, ADE might happen at sub-neutralizing antibody concentrations.

The existence of this effect indicates that the binding constants of viral antigenic epitopes to antibodies play a key role in determining whether an antibody will neutralize the virus or become an ADE trigger in complex with the virus.

There is evidence that the binding constant between the Fc region of an antibody and the Fc receptor of a phagocytic cell also plays an important role in promoting ADE. Antibody fucosylation lowers the FcγRIIIa/CD16a affinity (140). As was mentioned above patients with severe acute COVID-19 have increased concentration of antiviral IgGs that are afucosylated in their Fc region (141, 143, 144). These antibodies bind much better to CD16, and this enhanced binding probably facilitates antibody-mediated entry of SARS-CoV-2 cells into CD16+ immune cells, ultimately promoting infection of these cells (66).

##### How common ADE effects for COVID-19?

How common are antibodies that are capable of ADE of SARS-CoV-2 in the human population? ADE-capable antibodies were found in almost half of the acute COVID-19 patients (156) and in a significant proportion of convalescent plasmas of recovered patients (129). Among 93 plasma samples tested, 90 were capable of inducing ADEs *ex vivo* (74).

### Four scenarios of virus internalization by phagocytic cells

**Figure 6:**
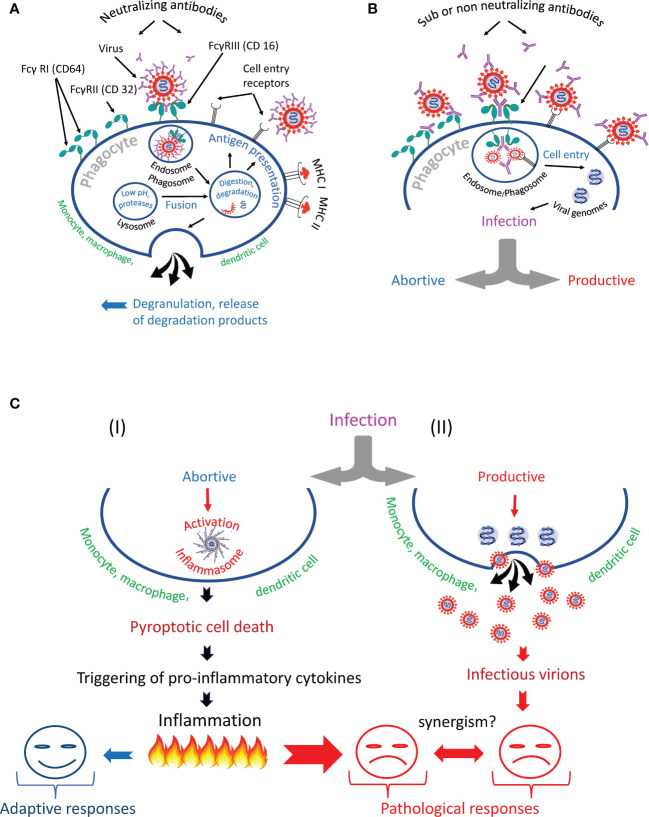
Antibody-dependent phagocytosis of SARS-CoV-2 in norm and pathology. **(A)** The phagocyte destroys the internationalized pathogen through the phagolysosomal pathway and presents its antigens *via* the major histocompatibility complexes (MHC I and MHC II). **(B)** The phagocyte engulfs but cannot inactivate SARS-CoV-2 and becomes infected. It is likely that the infection occurs due to the synergistic action of cell entry receptors and Fc receptors. **(C)** The virus escapes from the endosome/phagosome and the escape results in **(I)** abortive or **(II)** productive viral infection.

Summarizing the above information, we can identify several scenarios of phagocyte interaction with the virus (**Figure 6**). The first scenario involves the normal, natural course of events, designed by evolution to protect hosts from pathogens. The virus in complex with antibodies is taken up by the phagocyte *via* the Fc-receptor and enters the endosome/phagosome, which fuses with the lysosome to form the endo- or phagolysosome. During this fusion process, the virus is inactivated and destroyed, its proteins are used for antigen presentation (161) or released from the cell by degranulation (**Figure 6A**). The second scenario involves the survival of the virus (**Figure 6B**), which might result in the phagocyte infection (**Figure 6C**). In such a case, nature has developed a backup plan that programs the phagocytic cell to commit suicide by pyroptosis (I). This cell program prevents infection spread and attracts other immune cells to a site of infection. However, mass cell pyroptosis can cause uncontrolled inflammation, tissue damage, and severe complications of the disease. Alternatively, a productive virus infection of an immune cell can occur (II). Finally, trans-infection is possible. Such infection occurs when immune cells deliver a replication-competent virus as a parcel to the permissive cells. The harm to the host organism from all of these scenarios is obvious.

## Conclusions and perspectives

Available information indicates that SARS-CoV-2 is capable of infecting of phagocytic immune cells. There is evidence that professional phagocytes, such as monocytes, macrophages, and dendritic cells, as well as nonprofessional ones, such as B cells, can be targets of viral infection, which can be abortive or productive. Viral entry can be direct *via* cell-entry receptors or mediated by antibodies and Fc-receptors of immune cells. Most likely both types of receptors act synergistically and cooperate in helping the virus to infect a phagocytic immune cell.

In addition, trans-infection of target cells with SARS-CoV-2 virus has been demonstrated. The virus can attach itself to the receptors of an immune cell, travel with this cell as a passenger, maintaining the status of replication ability, reach the permissive target cell and infect it.

Regardless of how a virus enters immune cells or travels with them, it can cause pathological reactions. They can manifest themselves in the spread of a viral infection and/or in mass death of phagocytic cells *via* pyroptosis, accompanied by uncontrolled inflammatory cascades.

The mechanisms of penetration of replication-competent SARS-CoV-2 into immune cells deserve careful study because there are many blind spots related to the identification of cell receptors, proteases, and other molecules that may promote this process.

The ability of the virus to cause ADE by infecting immune cells should be particularly studied. SARS-CoV-2 most likely uses multiple mechanisms to enter cells *via* Fc receptors and cause enhanced inflammation. We also have a poor understanding of why some antibodies may contribute to ADE and some may not. Perhaps the difference in conformational stability of S protein epitopes (162), which can be facilitated by lowering pH during pathological development of COVID-19 (163–165) plays a role in increasing the probability of ADE. The role of afucosylated antibodies in the ADE process also deserves special attention.

Finally, the most important question that must be addressed is how ADE affects the severity of COVID-19.

## Author contributions

OM: Developing the idea of writing such a review, writing the main text, designing, and drawing illustrations. YN: Organizing regular manuscript discussions, conceptualization and reviewing the progress. DL: Writing the section “IgG afucosylation contributes to Fc-mediated virus phagocytic uptake, infection and disease severity, participation in regular discussion of the manuscript content. YY: Concept of introduction, outline of information presentation and design of **Figure 4**. JK: Concept and outline of information presentation, writing, the manuscript, design of tables. All authors contributed to the article and approved the submitted version.

## Funding

This research was funded by the Tomsk State University Development Programme (Priority 20-30), and by the Program of Fundamental Research in the Russian Federation for 2021–2030 period (project No. 121052600299-1).

## Acknowledgments

The authors are grateful to Dr. Dmitry Mazurov for his careful reading of the text and constructive criticism, which helped to significantly improve this review.

## Conflict of interest

Authors OM and DL was employed by Sendai Viralytics, LLC.

The remaining authors declare that the research was conducted in the absence of any commercial or financial relationships that could be construed as a potential conflict of interest.

## Publisher’s note

All claims expressed in this article are solely those of the authors and do not necessarily represent those of their affiliated organizations, or those of the publisher, the editors and the reviewers. Any product that may be evaluated in this article, or claim that may be made by its manufacturer, is not guaranteed or endorsed by the publisher.
