# Supplementary material for: SARS-CoV-2 infection of phagocytic immune cells and COVID-19 pathology: Antibody-dependent as well as independent cell entry
Source: Front Immunol. 2022 Dec 1;13:1050478. doi: 10.3389/fimmu.2022.1050478 (PMC9751203; doi:10.3389/fimmu.2022.1050478)
Supplement: Supplementary file 1 [file Table_1.docx]

**Supplemental Table 1. Evidence of immune cell infection.** The color with which the words are highlighted, or color of the font indicates the category to which the corresponding molecule, cell, or phenomenon belongs. Each color indicates one category. The titles of the articles in the first column that correspond to the information in the relevant row in the table are in blue.

| Title of Publication | Cohort | Immune  cells type | Finding | Model, material, organism, type of samples | Virus or viral molecule detection | Method of detection | Country,  place,  date of publication | Correlation with clinical symptoms. | Journal | frence  Ref. |
| --- | --- | --- | --- | --- | --- | --- | --- | --- | --- | --- |
| The Novel Severe Acute Respiratory Syndrome Coronavirus 2 (SARS-CoV-2) Directly Decimates Human Spleens and Lymph Node | Autopsies of 6 COVID-19 patients | Tissue resident CD68+ and CD169+ macrophages | Infected tissue-resident macrophages in spleens and lymph-nodes. | Autopsy | N-protein detection | Immuno-histochemistry | China,  Mar. 2020 | Yes | MedRxiv,  preprint | (61) |
| Circuits between infected macrophages and T cells in SARS-CoV-2 pneumonia | 88 patients with SARS-CoV-2-induced respiratory failure and 211 patients with pneumonia caused by other pathogens | Migratory and tissue resident alveolar macrophages (M2), which do not express ACE2, CCR7+  dendritic cells | SARS-CoV-2 transcripts in macrophages of 67% of samples from COVID-19 patients. In 38% of the samples, both plus and minus strands of SARS-CoV-2 were detected. | Bronchoalveolar lavage fluid samples from patients with COVID-19 | RNA products, plus and minus strands | Single-cell transcriptomic profiling | USA,  Jan. 2021 | Yes | Nature | (25) |
| Monocytes and macrophages, targets of SARS-CoV-2: the clue for Covid-19 immunoparalysis | 76 COVID-19 patients | Monocytes and monocyte derived M1 and M2 macrophages | Abortive infection of the immune cells. | Primary cell culture from COVID-19 patients and controls | RNA products | RT-PCR of infected cells | France,  Jan. 2021 | Yes | J Infect Dis | (69) |
| SARS-CoV-2-induced immune activation and death of monocyte-derived human circulating macrophages and dendritic cells | Healthy donors | Macrophages,  dendritic cells, and CD14+ monocytes. | Abortive viral infection of the immune cells. | Cell culture | N-protein | Flow cytometry, antibodies  against viral and N-protein | USA,  Iowa,  Mar. 2021 | N/A | J Infect Dis  . | (68) |
| Inflammasomes are activated in response to SARS-CoV-2 infection and are associated with COVID-19 severity in patients | 124 COVID-19 patients | Monocytes and macrophages | SARS-CoV-2 can infect human monocytes in vitro. Inflammasome activation in response to infection is associated with COVID-19 severity in patients. | Primary culture from healthy donors, in vitro infection of human monocytes, autopsy | RNA products, transcripts for N2, E, and RNase-P housekeeping gene | RT-PCR of infected cells | Brazil,  Mar. 2021 | Yes | J Exp Med  . | (71) |
| SARS-CoV-2 engages inflammasome and pyroptosis in human primary monocytes | 12 COVID-19 patients under intensive care and healthy donors | Primary monocytes | SARS-CoV-2 infection induces lytic cell death in human primary monocytes by triggering pyroptosis. | PBMCs from healthy donors and COVID-19 patients under intensive care. | Infectious virus RNA products | Quantitative RT-PCR,  SARS-CoV-2 virus titration | Brasil,  Mar. 2021 | Yes | Cell death discovery | (70) |
| Distinct uptake, amplification, and release of SARS-CoV-2 by M1 and M2 alveolar macrophages | None | M1 and M2  Macrophages  Monocytes | Alveolar macrophages are targets for productive SARS-CoV-2 infection. | Cell culture, transgenic mice, murine bronchial lavage fluid, human promonocytic THP-1, which represents human monocytes model. | RNA products,  negative-sense RNA  N-protein | RNAscope (green fluorescence-labeled probe against SARS-CoV-2 RNA), N-protein antibodies | China,  April 2021 | N/A | Cell Discovery | (67) |
| Pulmonary stromal expansion and intra-alveolar coagulation are primary causes of Covid-19 death | Autopsies of 12 COVID-19 patients | CD 68+ macrophages | The virus was replicating in the pneumocytes and macrophages. | Autopsy | RNA products- S- protein mRNA, S- an N-proteins | RNAScope and antibodies  against viral S and N-proteins | Sweden  and China,  May 2021 | Yes | Heliyon | (62) |
| Broad Severe Acute Respiratory Syndrome Coronavirus 2 Cell Tropism and Immunopathology in Lung Tissues From Fatal Coronavirus Disease 2019 | Autopsies of 5 COVID-19 patients | CD68+  or CD163+ macrophages, monocytes, neutrophils, natural killer (NK) cells, B cells, and T cells | Infiltrating immune cells were positive for viral proteins. | Autopsy | Viral structural and nonstructural proteins (S-, N-proteins, NSP8 and NSP13 proteins) | Immunofluorescence  staining, Immunohistochemistry | USA, Pittsburgh,  Jun. 2021 | Yes | J Infect Dis | (63) |
| SARS-CoV-2 infects human adipose tissue and elicits an inflammatory response consistent with severe COVID-19 | 8 autopsies | Adipose tissue macrophages CD45+CD14+ and CD11c- dendritic cells (ACE-2 negative) | Infection of adipose tissue macrophages induces inflammation; RNA accumulation. | Primary cell culture of adipose tissue of healthy donors and autopsies, single-cell RNA sequencing | RNA products, N-gene, N protein | RT-PCR of infected cells, flow cytometry | USA, Germany, Switzerland,  Oct. 2021 | Yes | *BioRxiv*  *preprint*  *and note in Clinical Infectious Disease* | (65) |
| Antibody-Dependent Enhancement of SARS-CoV-2 Infection of Human Immune Cells: In Vitro Assessment Provides Insight in COVID-19 Pathogenesis | Healthy donors and samples from three COVID-19 patients | Primary B cells, established cell culture of B-cells (Raji cells) monocytes, and macrophages | SARS-CoV-2 infection of primary B cells, macrophages, and monocytes, enhanced by convalescent serum from COVID-19 patients. ADE is demonstrated | Primary B cells, established cell culture | Infectious virus  Viral RNA, N protein | Virus titration, Flow cytometry, RT-PCR | China | Yes | Viruses | (72) |
| ACE2 can act as the secondary receptor in the FcγR-dependent ADE of SARS-CoV-2 infection | 93 convalescent plasma samples | Model cells transfected with CD32A gene and established cell culture of B-cells (Raji cells) | Among 93 plasma samples tested, 90 plasma exhibited canonical bell-shaped ADE curves, suggesting that most had the potential to cause ADE. Convalescent plasma from the severe groups mediates a stronger ADE effect than those from the mild group | Convalescent plasma samples, cell culture | Infectious pseudovirus | Model virus titration, flow cytometry and immunofluorescence | China,  Jan. 2022 | Yes | iScience | (74) |
| Antibody-dependent enhancement (ADE) of SARS-CoV-2 pseudoviral infection requires FcγRIIB and virus-antibody complex with bivalent interaction |  | FcγRIIB-expressing B-cells. Established cell culture of B-cells (Raji cells and **Daudi cell**) | Whereas the neutralizing non-capable ADE binds to RBD only in the “up” S-protein conformation state. ADE-capable antibodies bind to RBD in both “up” and “down” conformations. Such antibodies were called bivalent antibodies. | Cell culture | Infectious pseudovirus | Model virus titration, flow cytometry and immunofluorescence | China,  Jan. 2022 | N/A | Commun Biol  . | (75) |
| Inflammasome activation in infected macrophages drives COVID-19 pathology | Not indicated | CD16+ Macrophages | Viral replication in human macrophages triggers an inflammatory cascade and chronic viral infection in humanized mice. Blockade of inflammasome activation leads to the productive virus infection. | Cell culture, transgenic mice, autopsy | RNA products, genomic RNA and subgenomic viral RNA. Viral constructs that encode the green fluorescent protein | RT-PCR, Green fluorescent labeled protein,  flowcytometry | USA,  Brazil,  April 2022 | N/A | Nature | (22) |
| FcγR-mediated SARS-CoV-2 infection of monocytes activates inflammation | 9 healthy donors, from fresh plasma of 73 COVID-19 patients, plus frozen plasma samples from 60 COVID-19 patients, and samples from 5 autopsies | Monocytes CD16+, CD 64+, lung macrophages | 10% of blood monocytes in COVID-19 patients are infected with the virus. The infection is abortive. Monocyte infection ADE dependent. | Primary culture from healthy donors and COVID-19 patients, autopsies | RNA products, N-protein | Anti-double stranded RNA and N-protein antibodies | USA, UK,  Apr. 2022 | Yes | Nature | (66) |
| COVID-19 tissue atlases reveal SARS-CoV-2 pathology and cellular targets | 23 lung, 16 kidney, 16 liver and 19 heart COVID-19 samples from autopsies | LDB2 high  OSMAR high YAP1 high macrophages and CD14 high CD16 high inflammatory monocytes | Viral RNA presence in myeloid cells | Autopsy | RNA-products | Single cell sequencing | USA,  July 2022 | N/A | Nature | (26) |
| Infection of human lymphomononuclear cells by SARS-CoV-2 | 29 COVID-19 patients and 12 healthy controls | Monocytes, B and T lymphocytes | Productive virus infection of immune cells. | PBMCs from healthy donors and COVID-19 patients*,* autopsy | Infectious virus RNA products, double-stranded RNA, and mRNA of N-protein, | RT-PCR, cell, cytopathic effects, SARS-CoV-2 virus titration, flow cytometry and immunofluorescence | Brasil,  Aug. 2022 | N/A | Journal of Molecular Cell Biology  . | (64) |
| Sensing of SARS-CoV-2 by pDCs and their subsequent production of IFN-I contribute to macrophage-induced cytokine storm during COVID-19 | Healthy donors, the number of which is unknown | plasmacytoid dendritic cells, lung macrophages | Expression of viral proteins in pDC cells. Lung macrophages are infected with SARS-CoV-2 through phagocytosis of infected epithelial cells. | PBMCs from healthy donors, macrophages isolated from human lungs, Human alveolar epithelial cells | Infectious virus RNA products of N and E viral proteins | RT-PCR, SARS-CoV-2 virus titration, flow cytometry and immunofluorescence | USA, Sept. 2022 | N/A | Science | (73) |
| CiDRE+ M2c macrophages hijacked by SARS-CoV-2 cause COVID-19 severity | Samples from 122 hospitalized patients | M2c-type alveolar macrophages | Viral RNA accumulation in IL-10-induced M2c-type alveolar macrophages | Animal models, cell culture, human clinical samples, and primary cell cultures | RNA product of N gene | RT-PCR | Japan,  Oct.  2022 | Yes | *BioRxiv*  *preprint* | (92) |
